# Identification of Cx43 variants predisposing to ventricular fibrillation in the acute phase of ST-elevation myocardial infarction

**DOI:** 10.1093/europace/euac128

**Published:** 2022-08-09

**Authors:** Philippe Chevalier, Adrien Moreau, Francis Bessière, Sylvain Richard, Mohamed Chahine, Gilles Millat, Elodie Morel, Franck Paganelli, Nathalie Lesavre, Leslie Placide, François Montestruc, Bénédicte Ankou, Rosa Doñate Puertas, Babken Asatryan, Antoine Delinière, MAP-IDM Investigators

**Affiliations:** Université de Lyon, université Lyon 1, Inserm, CNRS, INMG, Lyon F-69008, France; Hospices Civils de Lyon, Groupement Hospitalier Est, Service de Rythmologie, Hôpital Cardiologique Louis Pradel, 59 Boulevard Pinel, 69677 Bron Cedex, France; PhyMedExp, INSERM U1046, CNRS UMR9214, Université de Montpellier, CHU Arnaud de Villeneuve, 34295 Montpellier, France; Université de Lyon, université Lyon 1, Inserm, CNRS, INMG, Lyon F-69008, France; Hospices Civils de Lyon, Groupement Hospitalier Est, Service de Rythmologie, Hôpital Cardiologique Louis Pradel, 59 Boulevard Pinel, 69677 Bron Cedex, France; PhyMedExp, INSERM U1046, CNRS UMR9214, Université de Montpellier, CHU Arnaud de Villeneuve, 34295 Montpellier, France; CERVO Brain Research Center, Quebec City, QC, Canada; Laboratoire de Cardiogénétique moléculaire, Centre de biologie et pathologie Est, Bron, France; Université de Lyon, université Lyon 1, Inserm, CNRS, INMG, Lyon F-69008, France; Hospices Civils de Lyon, Groupement Hospitalier Est, Service de Rythmologie, Hôpital Cardiologique Louis Pradel, 59 Boulevard Pinel, 69677 Bron Cedex, France; Service de cardiologie, Hôpital Nord, APHM, Marseille, France; Service de cardiologie, Hôpital Nord, APHM, Marseille, France; Université de Lyon, université Lyon 1, Inserm, CNRS, INMG, Lyon F-69008, France; Hospices Civils de Lyon, Groupement Hospitalier Est, Service de Rythmologie, Hôpital Cardiologique Louis Pradel, 59 Boulevard Pinel, 69677 Bron Cedex, France; eXYSTAT SAS, 4 rue Ernest Renan, 92240 Malakoff, France; Université de Lyon, université Lyon 1, Inserm, CNRS, INMG, Lyon F-69008, France; Hospices Civils de Lyon, Groupement Hospitalier Est, Service de Rythmologie, Hôpital Cardiologique Louis Pradel, 59 Boulevard Pinel, 69677 Bron Cedex, France; Signaling and Cardiovascular Pathophysiology—UMR-S 1180, Inserm, Université Paris-Saclay, Paris, France; Department of Cardiology, Inselspital, Bern University Hospital, Bern, Switzerland; Université de Lyon, université Lyon 1, Inserm, CNRS, INMG, Lyon F-69008, France; Hospices Civils de Lyon, Groupement Hospitalier Est, Service de Rythmologie, Hôpital Cardiologique Louis Pradel, 59 Boulevard Pinel, 69677 Bron Cedex, France

**Keywords:** Sudden cardiac death, Acute ventricular fibrillation, Connexin 43, ST-elevation myocardial infarction (STEMI), GJA1 variants

## Abstract

**Aims:**

Ventricular fibrillation (VF) occurring in the acute phase of ST-elevation myocardial infarction (STEMI) is the leading cause of sudden cardiac death worldwide. Several studies showed that reduced connexin 43 (Cx43) expression and reduced conduction velocity increase the risk of VF in acute myocardial infarction (MI). Furthermore, genetic background might predispose individuals to primary VF (PVF). The primary objective was to evaluate the presence of *GJA1* variants in STEMI patients. The secondary objective was to evaluate the arrhythmogenic impact of *GJA1* variants in STEMI patients with VF.

**Methods and results:**

The MAP-IDM prospective cohort study included 966 STEMI patients and was designed to identify genetic predisposition to VF. A total of 483 (50.0%) STEMI patients with PVF were included. The presence of *GJA1* variants increased the risk of VF in STEMI patients [from 49.1 to 70.8%, *P* = 0.0423; odds ratio (OR): 0.40; 95% confidence interval: 0.16–0.97; *P* = 0.04]. The risk of PVF decreased with beta-blocker intake (from 53.5 to 44.8%, *P* = 0.0085), atrial fibrillation (from 50.7 to 26.4%, *P* = 0.0022), and with left ventricular ejection fraction >50% (from 60.2 to 41.4%, *P* < 0.0001). Among 16 *GJA1* variants, three novel heterozygous missense variants were identified in three patients: V236I, H248R, and I327M. *In vitro* studies of these variants showed altered Cx43 localization and decreased cellular communication, mainly during acidosis.

**Conclusion:**

Connexin 43 variants are associated with increased VF susceptibility in STEMI patients. Restoring Cx43 function may be a potential therapeutic target to prevent PVF in patients with acute MI.

**Clinical trial registration:**

Clinical Trial Registration: https://clinicaltrials.gov/ct2/show/NCT00859300

## Graphical Abstract

**Graphical Abstract:**
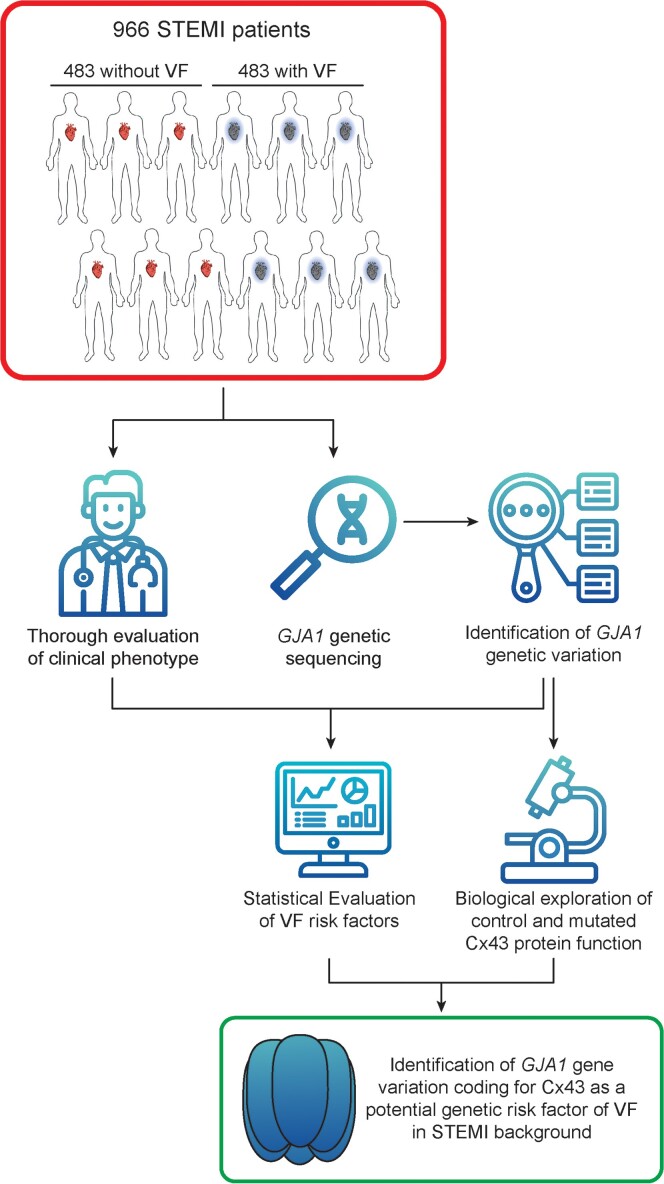

**What’s new?**

- Connexin 43 variants might be significant contributors to the risk of VF during acute MI.
- Connexin 43 may be a druggable target to prevent some ventricular fibrillation at the acute phase of myocardial infarction.
- Candidate gene/functional characterization approaches are valuable tools to identify novel potential genetic causes of diseases.

## Introduction

Sudden cardiac death (SCD) accounts for about 20% of all cardiovascular deaths in Europe, the USA, and worldwide.^1^ The main cause is related ST-elevation myocardial infarction (STEMI), and 3–10% of these patients suffer from ventricular fibrillation (VF) in the acute phase.^2^ Because myocardial infarction (MI) mostly happens at home, only 2% of patients receive emergency care on time. Primary VF (PVF) prevention is thus a major opportunity to reduce mortality from MI.

The normal myocardial rhythm is maintained by cell-to-cell transmission of electrical currents and molecules through gap junction (GJ) channels at the cell membrane and in the intercalated disks. Myocardial connection via GJ coupling decreases after MI and correlates with VF.^3^ The predominant ventricular GJ protein is connexin 43 (Cx43), encoded by the *GJA1* gene. Connexin 43 shows reduced expression and altered GJ localization after MI.^4^ Abnormal Cx43 function persists in the healed myocardium, conferring risk for VF and SCD over time.^5^

Several *in vivo* and *in vitro* studies have demonstrated the importance of Cx43 in myocardial function and VF occurrence.^5,6^ In the context of MI, myocardial cells are unable to connect and this creates a barrier to electric flow that causes arrhythmia. Targeted expression of Cx43 improved conduction velocity and reduced tachycardia susceptibility in an experimental MI model.^7^

A study in 17 families with oculodentodigital dysplasia identified genetic variants in *GJA1* (the gene encoding Cx43) and several cardiac abnormalities, including ventricular arrhythmias.^8^ Using GWAS, Bezzina and co-workers^9^ also suggested that genetic variants around genes regulating connexin cellular localization might be a risk factor of MI.

Nevertheless, the genetic basis of VF in the context of acute MI remains largely unknown. Identifying biomarkers that would reveal electrical risk during STEMI is therefore warranted.

We conducted the first prospective, multicentre study on the potential presence and impact of *GJA1* variants on VF susceptibility during the acute phase of STEMI. We hypothesized that patients with VF during MI might be predisposed because of *GJA1* gene variants affecting Cx43 protein function and/or localization. Such variants would increase the risk for developing VF during MI and even for SCD.

## Methods

A detailed Methods section can be found in the Supplementary Appendix.

### Trial oversight

The MAP-IDM (Identification of Molecular Markers of Sudden Death at the Acute Phase of Myocardial Infarction) trial was a multicentre, prospective, cohort study involving 43 centres in France, Belgium, and Switzerland. The two endpoints were to identify *GJA1* variants among STEMI patients and to analyse their association with PVF. All patients provided written informed consent prior to enrolment in the study. The study (NCT00859300) was approved by the relevant institutional review boards and was conducted in compliance with Good Clinical Practice guidelines and the principles of the Declaration of Helsinki.

### Patients

Patients aged ≥18 years who were admitted to an intensive care unit for MI and provided written informed consent were included in the study. Myocardial infarction was defined as the association of chest pain lasting at least 30 s, Q waves or ST elevation in two adjacent electrocardiogram (ECG) derivations, elevation of troponins (>1 µg/L), and a total coronary artery occlusion on coronary angiography. Patients with known medical history of acute coronary syndrome or cardiomyopathy and those who experienced VF during coronary catheterization were excluded.

### Trial procedures

The study prospectively included two groups: (i) patients with cardiac arrest who developed VF within 24 h post-MI, consistent with the time-definition of PVF,^10^ and (ii) patients with MI and no VF within the first 24 h post-MI. Demographic data, medical history, ongoing treatments, time to VF onset at inclusion, peak troponin value, culprit coronary artery, and left ventricular ejection fraction (LVEF) during hospitalization were recorded.

## Results

### Clinical parameters and ventricular fibrillation risk factors

Between December 2007 and August 2014, a total of 966 STEMI patients from 36 centres were included. Of them, 483 patients (50%) developed VF within the first 24 h of symptom onset, and the other 483 did not.

Univariate analysis revealed that age, sex, or smoking status did not affect the risk of developing VF. However, patients with beta-blocker intake (from 53.5 to 44.8%, *P* = 0.0085), patients with atrial fibrillation (from 73.6 to 50.7%, *P* = 0.0022), and patients with LVEF >50% (from 60.2 to 41.4%, *P* < 0.0001) had a reduced risk for developing VF in the first 24 h post-STEMI (*Tables 1* and *2*). Among the population with ECG data available, prolonged QRS duration, ST elevation, and RR were associated with an increased risk of VF, while the *P*-wave duration, PR interval, and QT duration had no impact on VF occurrence (*Tables 1* and *2*).

**Table 1:** Univariate and multivariate logistic regressions of ventricular fibrillation (rate of VF in subgroups)

Subgroup		VF	No VF	Univariate Odds ratio (95% CI)	*P*-value	Multivariate Odds ratio (95% CI) *P*-value
		No. of events (%)	Mean (SD)			
Age (*n* = 955)		57.6 (12.3)	56.7 (11.9)	1.03 (0.98–1.09)	0.25	Not included
Sex	Female (*n* = 179)	91 (50.8%)	88 (49.2%)			Not included
	Male (*n* = 778)	387 (49.7%)	391 (50.3%)	1.04 (0.75–1.45)	0.79	
Smoker	No (*n* = 410)	202 (49.3%)	208 (50.7%)			Not included
	Yes (*n* = 561)	267 (49.9%)	268 (50.1%)	0.97 (0.98–1.09)	0.85	
Beta-blocker intake	Yes (*n* = 397)	178 (44.8%)	219 (55.2%)			
	No (*n* = 561)	300 (53.5%)	261 (46.5%)	0.71 (0.55–0.92)	**0.0085**	0.76 (0.58–1.01) 0.061
Family History of SCD	Yes (*n* = 102)	44 (43.1%)	58 (56.9%)			Not included
	No (*n* = 842)	425 (50.5%)	417 (49.5%)	0.74 (0.49–1.13)	0.16	
BMI (*n* = 916)		26.2 (4.2)	26.7 (4.2)	1.15 (0.99–1.35)	0.07	Not included
Sinus Rhythm	Yes (*n* = 456)	231 (50.7%)	225 (49.3%)			
	No (*n* = 53)	14 (26.4%)	39 (73.6%)	0.37 (0.19–0.70)	**0.0022**	
LVEF (*n* = 944)		46.4 (11.4)	51.3 (10.6)	1.22 (1.15–1.30)	**<0**.**0001**	
LVEF (*n* = 944)	>50% (*n* = 539)	223 (41.4%)	316 (58.6%)			
	≤50% (*n* = 405)	244 (60.2%)	161 (39.8%)	0.47 (0.36–0.61)	**<0.0001**	0.44 (0.33–0.58) <0.0001
GJA1 variants	No (*n* = 839)	412 (49.1%)	427 (50.9%)			
	Yes (*n* = 24)	17 (70.8%)	7 (29.2%)	0.40 (0.16–0.97)	**0.0423**	0.42 (0.17–1.03) 0.0588
ECG: *P* (*n* = 430)		102.9 (14.5)	100.6 (16.0)	0.95 (0.90–1.01)	0.1255	Not included
ECG: PR (*n* = 453)		173.2 (31.0)	169.6 (26.2)	0.98 (0.95–1.01)	0.1833	Not included
ECG: QRS (*n* = 489)		100.7 (22.6)	95.0 (17.6)	0.93 (0.89–0.98)	**0**.**0028**	Not included (49% missing data)
ECG: QT (*n* = 506)		375.0 (55.2)	376.6 (46.8)	1.00 (0.99–1.02)	0.7261	Not included
ECG: susST (*n* = 507)		3.4 (2.7)	2.7 (1.8)	0.52 (0.34–0.77)	**0**.**0014**	Not included (48% missing data)
ECG: RR (*n* = 503)		760.2 (205.1)	830.0 (192.0)	1.01 (1.00–1.01)	**0**.**0001**	Not included (48% missing data)
Overall (*n* = 966)		483 (50.0%)	483 (50.0%)			

Bold values considered as statistically significant (*p* < 0.05).

CI, confidence interval; VF, ventricular fibrillation.

**Table 2:** Sensitivity analyses of *GJA1* variants on ventricular fibrillation in overall population and in male patients

	All patients *n* = 839/24 Odds ratio (95% CI)	*P*-value	Male patients *n* = 681/24 odds ratio (95% CI) *P*-value
Logistic regression (univariate analysis)	0.40 (0.16–0.97)	**0**.**04**	0.39 (0.16–0.95) **0.04**
Logistic regression (multivariate analysis)	*n* = 833/24 0.42 (0.17–1.03)	**0**.**06**	*n* = 676/24 0.39 (0.16–0.98) **0.045**
Exact conditional logistic regression (univariate analysis)	0.40 (0.14–1.02)	**0**.**06**	0.39 (0.13–0.999) **0.0497**
Exact conditional logistic regression (multivariate analysis)	*n* = 833/24 0.41 (0.14–1.10)	**0**.**08**	*n* = 676/24 0.40 (0.13–1.04) **0.06**
Propensity score using inverse probability of treatment weighting	0.47 (0.38–0.57)	**<0**.**001**	0.46 (0.37–0.57) **<0.001**
Propensity score using among the *GJA1* weighting	0.44 (0.13–1.44)	**0**.**18**	0.43 (0.13–1.40) **0.16**
Propensity score using matching	*n* = 300/24 0.39 (0.16–0.97)	**0**.**04**	*n* = 300/24 0.38 (0.15–0.94) **0.04**

Bold values considered as statistically significant (*p* < 0.05).

CI, confidence interval.

### Identification of novel *GJA1* missense variants

**Figure 1:**
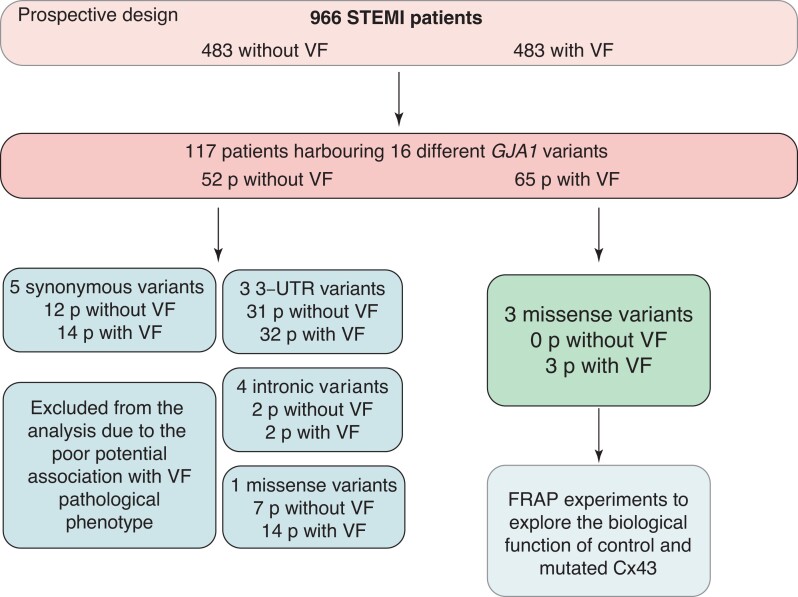
*GJA1* variant selection. Schematic representation of study workflow. Cx43, connexin 43; FRAP, fluorescence recovery after photobleaching; STEMI, ST-elevation myocardial infarction; VF, ventricular fibrillation.

Genetic sequencing was performed on the 966 STEMI patients to search for *GJA1* genetic variants, which were found in 117 (12%) patients (*Table 3*, *Figure 1*). Among the 117 STEMI patients with a *GJA1* variant, 65 (56%) experienced VF in the first 24 h post-MI (*Table 3*, *Figure 1*). Identified variants could be separated into four categories: (i) synonymous variants, (ii) variants targeting the 3′ UTR gene region, (iii) variants targeting introns, and (iv) exonic missense variants (*Figure 1*). To further analyse the implication of *GJA1* variants on the VF triggering susceptibility, we decided to exclude all *GJA1* variants except for exonic missense variants. Indeed, synonymous variants are not expected to play a role in the pathogenesis, and the potential pathogenic roles of 3′-UTR or profound intronic variants remain highly unclear. After applying these filters, 4 different genetic variants were found in 24 (2,5%) STEMI patients, among whom 17 (71%) suffered from VF within the first 24 h from symptom onset (*Table 3*).

**Table 3:** Identified *GJA1* variants

Variant type	dbSNP	Nucleotide change	Effect on protein	No. of patients without VF	No. of patients with VF
Synonymous	rs145215218	c.1128G > A	p.Arg376Arg	2	1
	rs201088822	c.411C > T	p.Tyr137Tyr	0	1
	rs201994305	c.432G > A	p.Lys144Lys	0	1
	rs57946868	c.717G > A	p.Arg239Arg	10	10
	rs766082259	c.612G > A	p.Thr204Thr	0	1
3’UTR	rs139128953	c.*243A > G	?	1	1
	rs397824185	c.1149 + 3lnsA (c*.3dup)	?	9	22
	rs72548744	c.*173G > A	?	21	9
Intronic	rs1013584065	c.−16-117A > G	?	0	1
	rs117533544	c.−16-65G > A	?	1	0
	rs56199702	c.−16-12T > A	?	0	1
	rs570422661	c.−16-71T > G	?	1	0
Exonic—missense	rs148384161	c.706G > A	p.Val236Ile	0	1
	rs17653265	c.758C > T	p.Ala253Val	7	14
	rs747365823	c.743A > G	p.His248Arg	0	1
	rs778383309	c.981C > G	p.Ile327Met	0	1
Subtotal				52	65
Total				117	

VF, ventricular fibrillation.

**Figure 2:**
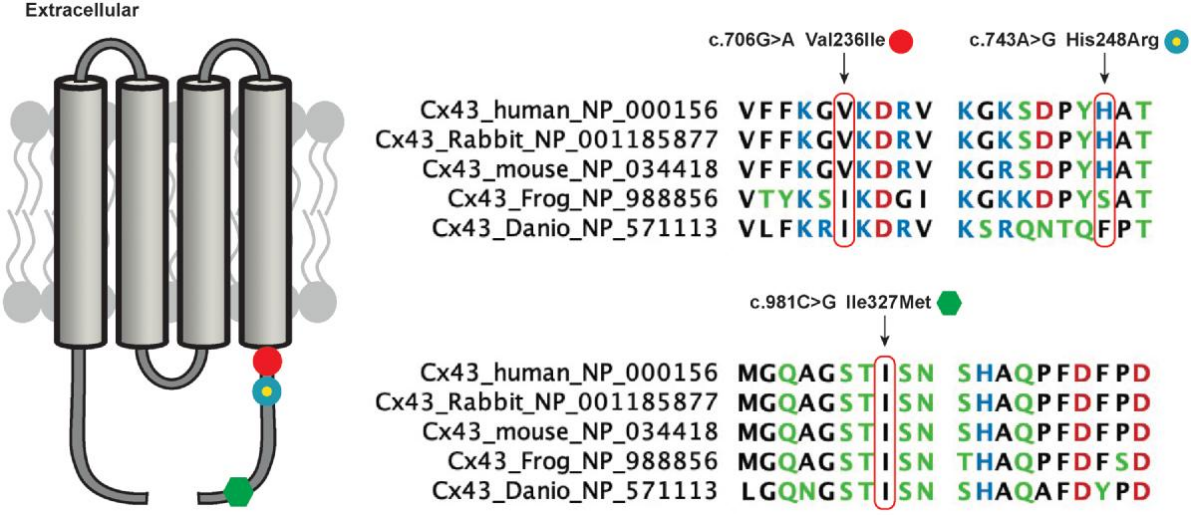
Identification of three novel *GJA1* missense variants. Left: Schematic 2D structure of the four transmembrane segments of human connexin 43 (Cx43) with the location of the three *GJA1* missense variants. Right: Multispecies protein sequence alignment illustrating the sequence conservation at the positions of the mutated residues.

Among these four *GJA1* variants observed in 24 patients, c.758C > T; p.Ala253Val (A253V) was identified in 21 patients (14 of whom experienced VF). The remaining three STEMI patients harbour three different novel heterozygous missense variants: c.706G > A; p.Val236Ile (V236I), c.743A > G; p.His248Arg (H248R), and c.981C > G; p.Ile327Met (I327M) (*Figure 2*). All patients experienced VF within the first 24 h from symptom onset (clinical details are provided in the supplementary section). Initial variant classification (based on the American College of Medical Genetics variant classification process) performed by a trained expert geneticist (G.M.) identified H248R and I327M as Class 3 variants and V236I as a Class 2 variant. The prevalence is: p.Val236Ile → ALL:0.011% – AFR:0.020% – EAS:0.0050% – SAS:0.0033% – NFE:0.018% – OTH:0.014%, p.His248Arg → ALL:0.00080% – SAS:0.0033% – NFE:0.00090%, and p.Ile327Met → ALL:0.0011% – AFR:0.0040% – NFE:0.0016%.

### Association between *GJA1* and ventricular fibrillation

Despite the low number of retained patients with *GJA1* missense variants (*n* = 24) in comparison with patients without missense *GJA1* variants (*n* = 839), the populations were similar with respect to age at MI and smoking status. Of note, women were absent from the *GJA1* variant group while they comprised 19% of the *GJA1* variant-free group. Patient characteristics according to *GJA1* status (presence or absence of a *GJA1* exonic missense variant) are summarized in *Table 4*. Interestingly, risk of VF decreased for patients without *GJA1* variants (from 70.8 to 49.1%, *P* = 0.0423; *Table 4*).

**Table 4:** Characteristics of the patients at baseline

Characteristic	No data for *GJA1* (*n* = 103)	*GJA1* variant (*n* = 24)	No *GJA1* variant (*n* = 839)	Total (*n* = 966)	Total missing
Median age (IQR), years	56 (48–64)	53 (49–64)	56 (49–65)	56 (49–65)	11
Female sex, *n* (%)	22 (23)	0 (0)	157 (19)	179 (19)	9
Smokers, *n* (%)	52 (55)	14 (58)	469 (57)	535 (57)	21
Median body mass index (IQR)^a^	25.9 (24.1–28.7)	26.7 (23.4–28.7)	25.9 (23.7–28.4)	25.9 (23.7–28.4)	50
Family history of SCD, *n* (%)	8 (8.5)	1 (4.2)	93 (11.3)	102 (10.8)	22
Beta-blocker intake, *n* (%)	49 (52)	7 (29)	341 (41)	397 (41)	8
Sinus rhythm, *n* (%)	47 (89)	14 (100)	395 (89)	456 (90)	457
Median LVEF (IQR)	50 (40–60)	48 (38–51)	50 (40–60)	50 (40–60)	22
Median ECG *P* (IQR)	100 (80–120)	100 (100–120)	100 (90–120)	100 (90–120)	536
Median ECG PR (IQR)	160 (160–200)	160 (160–180)	160 (160–180)	160 (160–180)	513
Median ECG QRS (IQR)	90 (80–100)	90 (80–110)	90 (80–110)	90 (80–110)	477
Median ECG QT (IQR)	360 (340–400)	400 (320–400)	360 (340–400)	360 (340–400)	460
Median ECG susST (IQR)	2 (1–3)	2.5 (1.5–4.5)	2.5 (1.5–4.0)	2.0 (1.5–4.0)	459
Median ECG RR (IQR)	760 (680–880)	760 (640–880)	760 (640–900)	760 (640–900)	463

ECG, electrocardiogram; IQR, interquartile range; LVEF, left ventricular ejection fraction; SCD, sudden cardiac death.

The body mass index is the weight in kilograms divided by the square of the height in metres.

In the multivariate model (*Table 1*), data were available for the three criteria retained in the model for 857 (88.7%) patients. One variable was associated with VF at the 5% level: LVEF >50% [odds ratio (OR): 0.44; 95% confidence interval (CI): 0.33–0.58; *P* < 0.0001]. Two additional variables were associated with VF at the 10% level: *GJA1* variants (OR: 0.42; 95% CI: 0.17–1.03; *P* = 0.059), and beta-blocker intake (OR: 0.76; 95% CI: 0.58–1.01; *P* = 0.061). Sensitivity analyses were concordant with the primary analysis with consistent OR (*Table 2*) and significant difference for propensity score using matching analysis. In the final multivariate logistic model, patients with beta-blockers intake, LVEF > 50%, and without *GJA1* variants had an estimated probability of VF of 19.5%. Reversely, patients without beta-blockers intake, LVEF ≤ 50%, and with *GJA1* variants had an estimated probability of VF of 63.3%. With a VF rate of 17/24 (70.8%) for patients with *GJA1* variants and 412/839 (49.1%) for patients without *GJA1* variants, the calculated a posteriori power to detect a *GJA1* effect was 56%.

In a *post hoc* exploratory analysis, we observed that the impact of *GJA1* variants was higher when LVEF increased. With LVEF >50%, we observed VF in 8/12 (66.7%) patients with *GJA1* variants compared with 177/192 (40.3%) patients without *GJA1* variants. The observed difference was 26.4%. This difference was 14.0% with LVEF ≤50%, with respective rates of 9/12 (75.0%) vs. 217/356 (61.0%), in patients with vs. without GJA1 variants.

In the male patient’s subgroup (*Table 2*), *GJA1* variants is significantly associated with VF (OR: 0.39; 95% CI: 0.16–0.98; *P* = 0.045) in multivariate analysis and also in propensity score using matching analysis.

### Connexin 43 missense variant biological exploration

Since the Cx43/A253V missense variant was found in STEMI patients both with and without VF, this variant is not expected to play a major role in the development of VF after STEMI. The biological study of Cx43 missense variants was thus focused on the three novel *GJA1* variants (V236I, H248R, and I327M) specifically found in STEMI patients suffering from VF.

### Connexin 43 cellular localization

**Figure 3:**
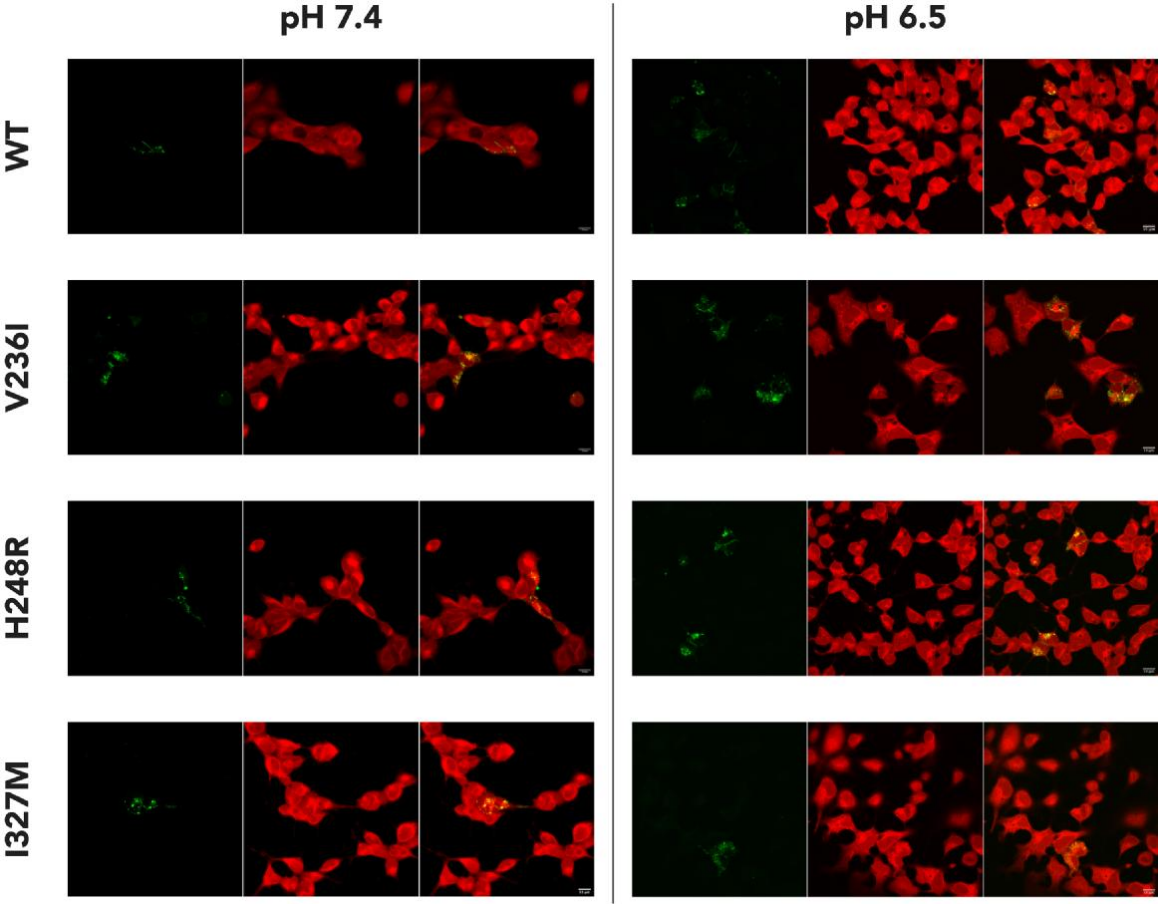
Wild-type (WT) and mutated connexin 43 (Cx43) cellular localization. Pictures of cells expressing WT or mutated (V236I, H248R, and I327M) hCx43 and loaded for 30 min with calcein. Mutants and the WT hCx43 were expressed as fusions to enhanced cyan fluorescent protein. For each panel, the three images from left to right show hCx43, the cells, and a merged image. The left side illustrates cells at physiologic (7.4) pH and right side illustrates cells at acidic (6.5) pH. Scale bars represent 15 µm.

The cellular distributions of transfected WT and mutated Cx43 were studied based on the fluorescence from enhanced cyan fluorescent protein fused to Cx43 constructs. Using transient HEK293 transfection, we observed that wild-type (WT) Cx43 was localized mainly to the cell surface. Indeed, structures forming gap–junction plaques at cell-to-cell contact areas were observed as a concentrated thin line at the cell border of cells expressing WT Cx43. In contrast, cells transfected with Cx43/V236I, Cx43/H248R, and Cx43/I327M revealed diffuse cytoplasmic Cx43 labelling that could be seen as intracellular spheres. These findings suggest that these three *GJA1* genetic variants might affect Cx43 trafficking and cellular localization. Changing the extracellular pH from 7.4 to 6.5 did not promote major changes in Cx43 cellular localization (*Figure 3*).

### Connexin 43 function

**Figure 4:**
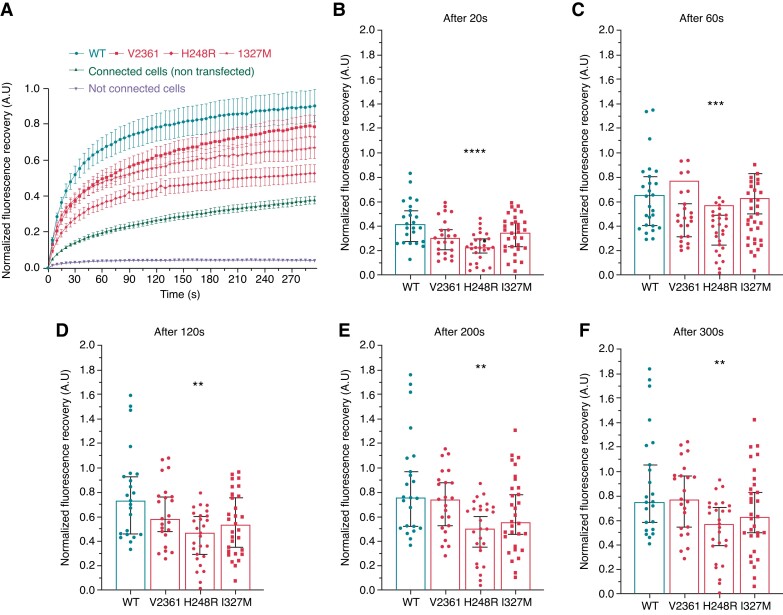
Functional properties of control and mutated hCx43 (V236I, H248R, and I327M) at physiological pH. (*A*) Mean normalized curves of fluorescence recovery after photobleaching are shown as a function of time (seconds), at physiological pH (7.4). Connected cells transfected with wild-type (WT) plasmids are shown in dark blue (circles). Connected cells transfected with mutated plasmids are shown in light blue (square: V236I, diamond: H248R, stars: I327M). Connected but not transfected cells are shown in grey and isolated cells are shown in black. Data are normalized using the WT condition. Data are shown as mean ± SEM. (*B–F*) Histograms showing focused results after 20 s (*B*), 60 s (*C*), 120 s (*D*), 200 s (*E*), and 300 s (*F*) after photobleaching. Histograms show the median (95% confidence interval). Statistical comparisons are made using a Kruskal–Wallis plus a Dunn’s test. Differences are deemed significant at *P < 0.05, **P < 0.01, ***P < 0.001, and ****P < 0.0001.

Connexin 43 function was assessed using the fluorescence recovery after photobleaching (FRAP) method, by bleaching a fluorescent calcein-loaded cell and recording the fluorescence recovery over time. Calcein is small enough (<1 kDa) to diffuse passively through GJs. Fluorescence recovery depends on the passage of calcein from a donor to the bleached cell. We evaluated Cx43 function in transiently transfected HEK293 cells in both physiological and acidic conditions, respectively resembling the myocardial environment before and after MI. At physiological pH, once bleached, isolated cells did not recover fluorescence (*Figure 4A*). HEK293 cells have endogenous, electrical-coupling protein expression, as demonstrated on recovery curves of connected but non-transfected cells (*Figure 4A*). Strong fluorescence recovery was observed in cells transfected with Cx43/WT, indicating efficient intercellular communication (*Figure 4A*). On the other hand, FRAP experiments with mutated Cx43 showed decreased fluorescence recovery when compared with cells transfected with Cx43/WT (*Figure 4A*). Fluorescence recovery after 20, 60, 120, 200, and 300 s post-bleaching was recorded to evaluate the timeline of intercellular communication (*Figure 4B–F*). The Cx43/H248R variant strongly decreased intercellular communication efficiency from the early to the end phase of fluorescence recovery (*Figure 4B–F*). Cells transfected with Cx43/WT were considered to have a complete recovery after 5 min (*Figure 4F*). For variants Cx43/V236I and Cx34/I327M, the loss of function at physiological pH was not statistically significant (*Figure 4*).

**Figure 5:**
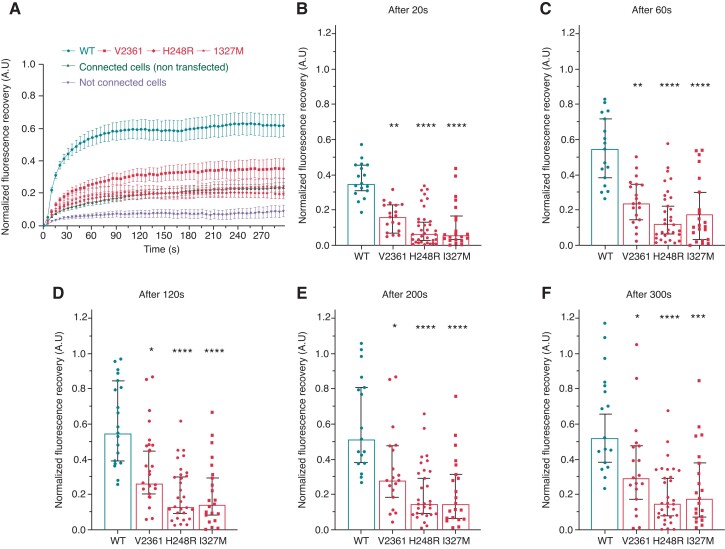
Functional properties of control and mutated hCx43 (V236I, H248R, and I327M) at acidic pH. (*A*) Mean normalized curves of fluorescence recovery after photobleaching are shown as a function of time (seconds), at acidic pH (6.5). Connected cells transfected with WT plasmids are shown in dark blue (circles). Connected cells transfected with mutated plasmids are shown in light blue (square: V236I, diamond: H248R, stars: I327M). Connected but not transfected cells are shown in grey and isolated cells are shown in black. Data are normalized using the WT condition. Data are shown as mean ± SEM. (*B–F*) Histograms showing focused results after 20 s (*B*), 60 s (*C*), 120 s (*D*), 200 s (*E*), and 300 s (*F*) after photobleaching. Histograms show the median (95% confidence interval). Statistical comparisons were made using a Kruskal Wallis plus a Dunn’s test. Differences were deemed significant at *P < 0.05, **P < 0.01, ***P < 0.001, and ****P < 0.0001.

Acidic pH (pH 6.5) was used to mimic acidosis, a characteristic of an ischaemic milieu. Accordingly, cells were incubated at acidic pH for at least 30 min before recordings were made. Cells transfected with Cx43/WT had a strong fluorescence recovery (*Figure 5*), although it was slower than at physiological pH (*Figure 4A*). This is consistent with reduced intercellular communication under acidic conditions. Evaluation of Cx43 variants at pH 6.5 revealed a loss of function for all three variants. The timeline of recovery indicated a strong decrease in intercellular communication efficiency from the early to the end phase of fluorescence recovery (*Figure 5A–F*).

## Discussion

To our knowledge, this is the first prospective designed study to identify *GJA1* genetic variants (encoding the Cx43 protein) in a large cohort of STEMI patients. Prior studies in patients suffering from PVF identified several rare variants in genes encoding a voltage-gated potassium channel (*KCNH2*, *HERG*)^11^ and the sodium channel alpha subunit (*SCN5A,* Na_V_1.5),^12^ supporting the idea that rare variants may predispose an asymptomatic population to VF. In the Arrhythmia Genetics in the NEtherlandS-AGNES study, it was demonstrated that familial sudden death occurred significantly more frequently among STEMI patients who experienced VF compared with STEMI patients who did not experienced VF.^13^ In the AGNES study, RR interval and ECG indices of conduction and repolarization during acute STEMI differ between patients who develop VF and patients who do not. However, the STEMI ECGs used in this study were retrieved retrospectively. Of note, the effects of the SNPs on ECG indices during an acute STEMI seemed to be similar in magnitude as those found in the general population, but the effects were too small to determine risk of VF. In the GEVAMI study from Denmark the authors found several sex differences in clinical characteristics and circumstances of arrest in women with low socioeconomic status.^14^ Details about ECG are not provided. In the Italian PREDESTINATION study, no information about ECGs was available but five independent predictors of primary VF were identified: familiarity, anterior MI, low systolic blood pressure, physical inactivity, and hypokalaemia.^15^ Prior studies identified Cx43 variant in atrial fibrillation background.^16^

Connexin 43 is the most abundant connexin in myocardial tissue and governs intercellular signalling communication.^17^ Several studies indicate that Cx43 may have a protective role in MI.^5–7,18^ In mouse models, loss of 50% of Cx43 expression increased the risk of ventricular tachycardia and VF.^19^ Increased expression of Cx43 in post-MI areas reduced the vulnerability to ventricular arrhythmia.^7,20^ In mice, lentivirus-mediated delivery of Cx43 into acute myocardial lesions conferred a long-lasting antiarrhythmic effect.^5^ Short-term enhancement of GJ coupling during acute MI produced more homogeneous infarct scars, reducing late susceptibility to post-MI arrhythmias.^18^ After MI, the density of Cx43 and myocardial conduction velocity decrease.^21^ During ischaemia, GJs partially close, and Cx43 is dephosphorylated and moves away from the GJs.^22^ Uncoupling of the GJs slows conduction and can lead to re-entry and subsequent VF.^21,23^ After uncoupling, transfer of survival factors that protect the myocardial cells is hampered. In mouse models expressing a modified Cx43 with a truncated regulatory CT terminal region (K258stop/KO mice), there is a predisposition to arrhythmias after experimental coronary occlusion. The K258stop/KO variant abrogates chemical regulation of Cx43, including response to acidic pH and phosphorylation sites. Our own previous studies showed that Cx43 dephosphorylation at S282 triggers arrhythmias and contributes to cardiomyocyte death upon ischaemia-reperfusion (I-R) by activating the p38/Fas/FADD pathway, providing a novel molecular mechanism and potential target for protecting against cardiac I-R injury.

In the present study, four missense *GJA1* variants were identified in 17 (3.5%) of 483 STEMI patients with VF. The three measured missense variants directly affected Cx43 function *in vitro*, as intercellular communication was impeded and delayed, especially under acidic conditions that mimic MI. Statistically, we were also able to establish an association between *GJA1* variants and VF risk in patients with STEMI. The decrease in presence at the membrane is not sufficient (for these mutations) to induce a significant loss of function (loss of communication). This is also probably why there are no major problems with cardiac cell communication apart from ischaemia, therefore no disease. Nevertheless, cellular acidification is a process already known to cause connexin closure. Thus, by combining the reduction in the presence of the membrane and the closure due to ischaemia, this ended up with the very significant loss of function observed with the variants.

Recent investigations in dogs have shown that prolonged QRS during coronary occlusion is related to lower collateral flow.^7^ In our study, prolonged QRS duration was significantly associated with an increased risk of VF, pointing towards abnormal myocardial conduction as a critical prerequisite for ischaemic VF. We found that there was no clear genotype–phenotype association because of the low number of patients with both available ECG and *GJA1* variants. Consequently, the association between QRS duration and variant carriers could not be quantified.

Previous bioinformatic analysis has shown that Cx43 has a role in MI.^9^ Both the H248R and the V236I variants have been potentially associated with oculodentodigital dysplasia (H248R, variant of unknown significance) and hypoplastic left heart syndrome 1, syndactyly type 3, oculodentodigital dysplasia (V236I). Of note, the trained expert geneticist indicated that due to the highest novelty of the potential genotype/phenotype associations, he would have classified all variants as Class 3 (variants of unknown significance).

### Study limitations

Because of missing data in the multivariate analyses and the small number of patients with *GJA1* variants, the a posteriori power (56%) was less than expected (80%). It is also known that patients who suffer SCD very often have several contributing factors. In this study, the phenotype of PVF was prospectively strictly defined. We could not identify Cx43 variants in all patients that suffered VF after STEMI, indicating that Cx43 is likely one of multiple unrecognized genetic risk factors for VF complicating STEMI. This study would also benefit from a replication study specially to confirm the *P*-value obtained with the multiple confounding clinical factors. We agree that it is an important issue and that a confirmatory study will add value to our results. However, we did not found any available data base with the same patient phenotype. The genetic basis of VF is likely to comprise multiple genes rather than one single gene. The gene candidate approach might be taken with cautious due to the high polymorphism of the human genome. Nevertheless, *GJA1* variants may help to identify a previously unknown population at risk for VF post-MI. The functional study of Cx43 variants is based on fluorescence transfer between cells, one great implement would be to measure the real Cx43 conductance using double patch-clamp experiments and also to implement more sophisticated cell model. Also, functional studies are focused on cell coupling while other non-canonical Cx43 roles have been described.^24^ Further research in the area of genetic contributors to ischaemia-related VF risk will shed light on the complex genetic architecture of MI and identify molecular targets for precision therapy. In our study, consanguinity was not assessed. Consanguinity as a risk factor for VF during MI has been extensively discussed,^9,25,26^ although with limited progress in the identification of the fundamental molecular and genetic determinants.

## Conclusions

Our findings support Cx43 variants as significant contributors to the risk of VF during acute MI. Although the genetic architecture of VF is likely complex, our results encourage the use of a candidate gene approach to better understand the underlying genetic background of VF.

### Perspectives

The MAP-IDM study provides:

- A better understanding of electrical instability during acute myocardial ischaemia.
- A better identification of patients at risk of sudden death in patients with coronary heart disease.
- New therapeutic target in a field where pharmaceutical intervention is weak.

## Supplementary material

Supplementary material is available at *Europace* online.

## Supplementary Material

euac128_Supplementary_Data

## Data Availability

The data that support the findings of this study are available from the corresponding author upon reasonable request.
