# Supplementary material for: Identification of Cx43 variants predisposing to ventricular fibrillation in the acute phase of ST-elevation myocardial infarction
Source: Europace. 2022 Aug 9;25(1):101–11. doi: 10.1093/europace/euac128 (PMC10103570; doi:10.1093/europace/euac128)
Supplement: euac128_Supplementary_Data [file euac128_supplementary_data.docx]

**Identification of Cx43 variants predisposing to ventricular fibrillation in the acute phase of ST-elevation myocardial infarction**

**Supplementary appendix**

[List of investigators 2](#_Toc61898226)

[Supplementary methods 4](#_Toc61898227)

[Genetic analysis 4](#_Toc61898228)

[Bioinformatic analysis 4](#_Toc61898229)

[Statistical analysis for the predictors for SCD 4](#_Toc61898230)

[Mutagenesis 5](#_Toc61898231)

[Cell culture 5](#_Toc61898232)

[Fluorescence recovery after photobleaching (FRAP) 6](#_Toc61898233)

[Supplementary Results: 7](#_Toc61898234)

[Patient 1: 7](#_Toc61898235)

[Patient 2: 7](#_Toc61898236)

[Patient 3: 7](#_Toc61898237)

[Supplementary References 8](#_Toc61898238)

# List of investigators

Pr Franck Paganelli, Dr Nathalie Lesavre, Service de cardiologie, Hôpital Nord, *APHM Marseille*;

Pr Gérald Roul, Unité de soins intensifs en cardiologie, Nouvel Hôpital civil Strasbourg, *Strasbourg*;

Pr Philippe Chevalier, Service rythmologie, Chevalier, *CHU Lyon*;

Pr Pierre Yves Gueugniaud, Service d'anesthésie-réanimation I, *CHU Lyon-Sud*;

Pr Pierre Coste, Dr Edouard Gerbaud, Hôpital Cardiologique, Service de soin intensif cardiologique, *CHU Bordeaux*;

Dr Antoine Dompnier, service de cardiologie, *CH Annecy*;

Pr Florence Leclercq, Hôpital Arnaud de Villeneuve, service soins intensifs cardiologie, *CHU Montpellier*;

Dr Jean-Sylvain Hermida, service cardiologie, *CHU Amiens* ; Dr Geneviève Jarry, service de soins intensifs cardiologiques, CHU Amiens;

Dr Michel Lopez, Unité de soins intensifs et de cardiologie, *Hôpital Saint-Joseph Saint-Luc Lyon*;

Dr Alain Cariou, Dr Virginie Lemiale, Service de réanimation médicale, Hôpital Cochin, APHP *Paris*;

Dr Dominique Babuty, Hôpital Trousseau, Service de cardiologie, *CHRU Tours;*

Dr Jacques Mansourati, Dr Pascale Quiniou, Hôpital de la Cavale Blanche, service de cardiologie, *CH Brest*;

Dr Vincent Probst, Service de cardiologie, Hôpital Laennec, *CHU Nantes*;

Dr Paul Bru, Service de cardiologie, *CH La Rochelle*;

Dr Patrick Evrard, Dr Sandrina Bouhon, service soins intensifs, *Cliniques Universitaires de Mont Godinne, Yvoir-Namur, Belgique*;

Pr Jean-Claude Deharo, Dr Jean-Philippe Mouret, Hôpital de la Timone, service de cardiologie, *APHM*;

Dr Philippe Maury, Hôpital Rangueil, service de cardiologie, *CHU Toulouse*;

Dr Florent Briand, Pr François Schiele, Unité de soins intensifs de cardiologie, *Hôpital J. Minjoz, Besançon*;

Dr Jean Sacrez, Clinique Saint- Pierre, Unité de cardiologie médicale et rythmologie interventionnelle *Perpignan*;

Dr Gaël Clerici, Service de cardiologie , groupe hospitalier Sud Réunion, *Saint Pierre de La Réunion*;

Dr Benaïssa Agraou, Service cardiologie, *CH Valenciennes;*

Dr Lionel Beck, Dr Pierre François Winum, service cardiologie, *CHU Nîmes*;

Dr Franck Chemouni, Hôpital intercommunal - Service de cardiologie, *Lagny sur Marne*;

Dr Jean-Pierre Monassier, Dr Jean-Yves Wiedeman, Unité de soins intensifs de cardiologie, *Hôpital Emile Müller, Mulhouse*;

Dr Xavier Lamit, Unité de soins intensifs en cardiologie, CH intercommunal de Fréjus St Raphael, *Fréjus*;

Pr Jean Sylvain Ponsonnaille, Dr Hassan Mansour, Hôpital Gabriel Montpied, Service de cardiologie et maladies vasculaires, *CHU Clermont-Ferrand*;

Dr Alain Gressard, Dr Cyril Besnard, Service de cardiologie et affections vasculaires, *Hôpital Croix Rousse CHU Lyon*;

Dr Isabelle Labioche, Dr Xavier Tabone, service de cardiologie, Centre hospitalier Jacques Cœur, *CH Bourges*;

Dr Anne-Sophie Rieschner, Dr Sébastien Buffler, service cardiologie, *CH Haguenau*;

Dr Patrick Buttard, service de cardiologie et soins intensifs *CH* William Morey *Châlon-sur-Saône*;

Pr Jean-Pierre Camous, Pr Emile Ferrari, Hôpital Pasteur, USIC - Service de cardiologie, *CHU Nice*;

Dr Christophe Prost, Service de cardiologie et soins intensifs, *CH Vienne*;

Dr Julien Laborderie, service de soins intensifs, Centre Hospitalier de la Côte Basque, *CH Bayonne*;

Dr Saïd Ahres, Service de cardiologie, Hôpitaux Civils de Colmar*;*

Pr Philippe Asseman, hôpital cardiologique, service d'urgence, soins intensifs et réanimation cardiovasculaire, *CHRU Lille*;

Dr Patrick Bert-Marcaz*, service de cardiologie, CH Mâcon*;

Dr Vincent Malquarti, Centre cardiologique de l'ouest lyonnais, *Clinique de la Sauvegarde, Lyon*;

Dr Yves Cottin, Service cardiologie et soins intensifs, Bocage Central, *CHRU Dijon*;

Dr Pierre Laurent, Hôpital d'instruction des armées Ste Anne, service cardiologie*, Toulon*;

Dr Haran Burri, Service cardiologie, *Hôpitaux universitaire de Genève, Suisse.*

# Supplementary methods

## Genetic analysis

Genomic DNA samples were screened for *GJA1* (NM_000165) variants by Sanger sequencing of amplicons using PCR primers. Such primers were designed to specifically target the coding region of *GJA1*, which is composed of 2 exons (the first is untranslated and the second has 1149 bp of coding sequence). All amplifications were performed using HotStarTaq^®^ DNA Polymerase (Qiagen). PCR products were purified and sequenced on both strands by conventional dideoxy sequencing using the BigDye^®^ Terminator v.3.1 Cycle Sequencing Kit (Life Technologies) on an ABI Prism 3130 XL DNA analyzer (Life Technologies).

## Bioinformatic analysis

The detected gene variants (i.e., missense, synonymous substitution, and deletion) were further analyzed for pathogenicity using filtering steps. Only missense variants in patients with VF were retained. The clinical significance of the variants was assessed using the information about previous reports in ClinVar (<https://www.ncbi.nlm.nih.gov/clinvar/>) and the variant frequencies in the Genome Aggregation Database (<https://gnomad.broadinstitute.org/>). The functional impact and protein domains affected by single nucleotide changes of each missense variant were evaluated in silico using bioinformatic prediction tools PolyPhen2,^1^ SIFT,^2^ and MutationTaster.^3^

## *Statistical analysis for the predictors for SCD*

A sample size of 1000 patients were needed to evaluate the arrhythmogenic impact of *GJA1* polymorphisms in patients with PVF, with a 80% power to detect a 25% lower risk of SCD, at a two-sided significance level of 5%, under the assumption of an 50% event rate for the absence of GJA1 polymorphisms patients. Incidence rate of 25/1000 (2.5%) of *GJA1* polymorphisms was considered and this disbalance was has been taken into account in the sample size calculation.

Continuous variables are presented as means and standard deviations (SD), or medians and interquartile ranges. Categorical variables are provided with percentages. Univariate logistic regression analysis was performed to identify predictors for SCD. Variables with significant predictive potential (i.e. p<0.05) in the univariate analysis with <20% of missing data were included in a multiple logistic-regression analysis to search for independent predictors. Subsequently, a posteriori power was calculated for exploratory purposes. Model calibration was assessed with the use of the Hosmer–Lemeshow test. Odds ratios (OR) were reported with 95% two-sided confidence intervals (CIs).

Sensitivity analyses were performed using exact logistic regression, to handle the few patients with *GJA1* polymorphisms and the disbalance in sample size, and propensity score to limit the influence of potential confounding variables. For computation of the propensity scores, a logistic regression with SCD as the outcome and potential confounding covariates (age, smoking status at the time of MI, family history of VF, body mass index [BMI], LVEF, and beta-blockers therapy) were included. Missing values were not replaced and variables with more than 20% of missing data were not included in the propensity score. All analyses were performed with the use of SAS^®^ software, version 9.4.

## Mutagenesis

Mutants hCX43 were generated by Q5 Site-Directed Mutagenesis Kit according to the manufacturer's instructions (New England Biolabs Inc., Canada). The hCX43/mutants were constructed from WT channels (NCBI accession number: NM_000165) using the following mutagenic forward and reverse primers (mutated sites are bold, and codons are underlined):

| hCx43-V236I-F | CTTCAAGGGC**A**TTAAGGATCG |
| --- | --- |
| hCx43-V236I-R | AAAACATAGAAGAGTTCAATGATATTC |
| hCx43-H248R-F | GACCCTTACC**G**TGCGACCAGT |
| hCx43- H248R-R | GCTCTTTCCCTTAACCCGATC |
| hCx43-I327M-F | GAAGCACCAT**G**TCTAACTCCCATGC |
| hCx43-I327M-R | CCGCCTGCCCCATTCGAT |

Mutants and the wild-type (WT) hCX43 cloned into pECFP-N1 vector (BD Biosciences Clontech) a generous gift from Michael Gollob (University of Toronto, Ontario, Canada) expressed as fusions to ECFP. Mutant and the wild-type (WT) hCX43 in a pECFP-N1 constructs were purified using EZ-10 Spin Column Plasmid DNA Minipreps Kit (Biobasic, Canada).

## Cell culture

Human embryonic kidney HEK-293 (HEK293) cells were transfected with complementary DNA from wild type or mutant human hCx43 (hCx43/V236I, hCx43/H248R, and hCx43/I327M) using the calcium-phosphate method Huang et al.^4^. Cells were grown at 37°C in 5% CO_2_ humidified atmosphere in high-glucose Dulbecco's Modified Eagle Medium supplemented with 10% fetal bovine serum, 100 U/mL penicillin G, and 10 mg/mL streptomycin (GIBCO-BRL Life Technologies). The human Cx43 constructs were inserted in pECFP-N1. During experiments, ECFP fluorescent cells were identified as transfected cells. ECFP is excited at 434 nm and emits fluorescence at 477 nm.

## Fluorescence recovery after photobleaching (FRAP)

Between 24 to 72 h post-transfections, cells were loaded for 30 minutes at 37°C, using 2 µmol/L of calcein red-orange-AM (CellTrace^TM^, Thermofisher Scientific). The extracellular solution used for loading at pH 7.4 (in mmol/L) consisted of 154 NaCl, 5.6 KCl, 2 CaCl_2_, 1 MgCl_2_, 8 glucose, 10 HEPES; and the one used for loading at pH 6.5 (in mmol/L) consisted of 154 NaCl, 5.6 KCl, 2 CaCl_2_, 1 MgCl_2_, 8 glucose, and 10 MES. Calcein red-orange is excited at 577nm and emits fluorescence at 590 nm. Experiments were performed in a confocal microscope (upright and inverted, LSM 880 and 800, Zeiss) equipped with a 40x (water immersion) objective and appropriate lasers. Before each experiment, a snapshot was taken with both ECFP and calcein red labelling to visualize transfected WT or mutated connexins and calcein red-orange. For FRAP experiments, 63 images of calcein red-orange fluorescence were recorded every 5 seconds. After the 3^rd^ image, selected cells were bleached with high intensity 561nm laser stimulation. The fluorescence recovery was studied as the mean fluorescence intensity of bleached areas. Data were analyzed using custom-written Matlab scripts. A first analysis round was realized to identify the maximal recovery value using a bi-exponential fit: A * (1-A_Fast_*exp(-t/Tau_Fast_) - A_Slow_ * exp(-t/Tau_Slow_)). The mean of the maximal recovery was determined for WT and was used to normalize all conditions tested.

# Supplementary Results:

## Patient 1:

A man, 61 years old, was admitted at the hospital for a ST-elevated myocardial infarction. He has no familial sudden cardiac death history and no specific treatment (statin, ß-blocker, insulin and sulfamid). He was non-smoker. His left ventricular ejection fraction was 70%. Blood testing indicated a troponin pic at 18.6 µg/l. The genetic study revealed a *GJA1* nucleotide substitution (c.706G>A) causing the Val236Ile missense variation on the hCx43 protein.

## Patient 2:

A man, 53 years old, was admitted at the hospital for a ST-elevated myocardial infarction. He has no familial sudden cardiac death history. He took Deroxat® and Temesta® but was free of statin, ß-blocker, insulin and sulfamid. He was non-smoker. His left ventricular ejection fraction was 56%. Blood testing indicated a troponin pic at 10.7 µg/l. The genetic study revealed a *GJA1* nucleotide substitution (c.743A>G) causing the His248Arg missense variation on the hCx43 protein.

## Patient 3:

A man, 74 years old, was admitted at the hospital for a ST-elevated myocardial infarction. He has no familial sudden cardiac death history. He took Bipretax® and insulin but was free of statin, ß-blocker and sulfamid. He was non-smoker. His left ventricular ejection fraction was 56%. Blood testing indicated a troponin pic at 10.7 µg/l. He showed 2^nd^ degree atrioventricular block. ECG parameters were: QRS: 110 ms, QT: 400ms, sus-ST elevation of 4.5mm with RR interval of 800ms. The genetic study revealed a *GJA1* nucleotide substitution (c.981C>G) causing the Ile327Met missense variation on the hCx43 protein.

Supplementary table 1 :

| **Table 3. Characteristics of the patients at baseline.** | | | | | |
| --- | --- | --- | --- | --- | --- |
| **Characteristic** | **No data for *GJA1***  **(N = 103)** | ***GJA1* variant**  **(N = 24)** | **No *GJA1* variant**  **(N = 839)** | **Total**  **(N = 966)** | **Total Missing** |
| Median age (IQR), years | 56 (48–64) | 53 (49–64) | 56 (49–65) | 56 (49–65) | 11 |
| Female sex, no. (%) | 22 (23) | 0 (0) | 157 (19) | 179 (19) | 9 |
| Smokers, no. (%) | 52 (55) | 14 (58) | 469 (57) | 535 (57) | 21 |
| Median body mass index (IQR)† | 25.9 (24.1–28.7) | 26.7 (23.4–28.7) | 25.9 (23.7–28.4) | 25.9 (23.7–28.4) | 50 |
| Family History of SCD, no. (%) | 8 (8.5) | 1 (4.2) | 93 (11.3) | 102 (10.8) | 22 |
| Beta-blocker intake, no. (%) | 49 (52) | 7 (29) | 341 (41) | 397 (41) | 8 |
| Sinus rhythm, no. (%) | 47 (89) | 14 (100) | 395 (89) | 456 (90) | 457 |
| Median FEVG (IQR) | 50 (40–60) | 48 (38–51) | 50 (40–60) | 50 (40–60) | 22 |
| Median ECG P (IQR) | 100 (80–120) | 100 (100–120) | 100 (90–120) | 100 (90–120) | 536 |
| Median ECG PR (IQR) | 160 (160–200) | 160 (160–180) | 160 (160–180) | 160 (160–180) | 513 |
| Median ECG QRS (IQR) | 90 (80–100) | 90 (80–110) | 90 (80–110) | 90 (80–110) | 477 |
| Median ECG QT (IQR) | 360 (340–400) | 400 (320–400) | 360 (340–400) | 360 (340–400) | 460 |
| Median ECG susST (IQR) | 2 (1–3) | 2.5 (1.5–4.5) | 2.5 (1.5–4.0) | 2.0 (1.5–4.0) | 459 |
| Median ECG RR (IQR) | 760 (680–880) | 760 (640–880) | 760 (640–900) | 760 (640–900) | 463 |

† The body mass index is the weight in kilograms divided by the square of the height in meters.

# Supplementary References

1. Adzhubei IA, Schmidt S, Peshkin L, et al. A method and server for predicting damaging missense mutations. Nat Methods 2010;7(4):248–9.

2. Kumar P, Henikoff S, Ng PC. Predicting the effects of coding non-synonymous variants on protein function using the SIFT algorithm. Nat Protoc 2009;4(7):1073–81.

3. Schwarz JM, Rödelsperger C, Schuelke M, Seelow D. MutationTaster evaluates disease-causing potential of sequence alterations. Nat Methods 2010;7(8):575–6.

4 Huang H, Priori SG, Napolitano C, O'Leary ME, Chahine M. Y1767C, A novel *SCN5A* mutation, induces a persistent Na^+^ current and potentiates ranolazine inhibition of Na_v_1.5 channels. Am J Physiol Heart Circ Physiol. 2011;300(1):H288-H299. doi:10.1152/ajpheart.00539.2010
